# SSM-Net: Enhancing Compressed Sensing Image Reconstruction with Mamba Architecture and Fast Iterative Shrinking Threshold Algorithm Optimization

**DOI:** 10.3390/s25041026

**Published:** 2025-02-09

**Authors:** Xianwei Gao, Bi Chen, Xiang Yao, Ye Yuan

**Affiliations:** Beijing Electronic Science and Technology Institute, Beijing 100070, China; gaoxianwei@besti.edu.cn (X.G.); 20232940@mail.besti.edu.cn (B.C.); 20233910@mail.besti.edu.cn (X.Y.)

**Keywords:** compressive sensing, image reconstruction, Mamba, state-space modeling, FISTA

## Abstract

Compressed sensing (CS) is a powerful technique that can reduce data size while maintaining high reconstruction quality, which makes it particularly valuable in high-dimensional image applications. However, many existing methods have difficulty balancing reconstruction accuracy, computational efficiency, and fast convergence. To address these challenges, this paper proposes SSM-Net, a novel framework that combines the state-space modeling (SSM) of the Mamba architecture with the fast iterative shrinking threshold algorithm (FISTA). The Mamba-based SSM module can effectively capture local and global dependencies with linear computational complexity and significantly reduces the computation time compared to Transformer-based methods. In addition, the momentum update inspired by FISTA improves the convergence speed during deep iterative reconstruction. SSM-Net features a lightweight sampling module for efficient data compression, an initial reconstruction module for fast approximation, and a deep reconstruction module for iterative refinement. Extensive experiments on various benchmark datasets show that SSM-Net achieves state-of-the-art reconstruction performance while reducing both training and inference reconstruction time, making SSM-Net a scalable and practical solution for real-time applications of compressed sensing.

## 1. Introduction

Compressed sensing (CS) has gained prominence in signal processing due to its ability to reconstruct high-dimensional signals from significantly fewer measurements [1]. This technique proves especially effective in scenarios where resources for data acquisition, such as bandwidth and storage, are constrained [2,3,4,5,6,7]. By exploiting the inherent sparsity or compressibility of signals, CS offers a structured approach to signal recovery applicable across various fields, including medical imaging, telecommunications, and industrial automation.

CS works in two main steps. First, during the sampling phase, a high-dimensional signal $x\in R^{n}$ is projected into a lower-dimensional measurement $y\in R^{m}$ (where m≪n) using a sensing matrix Φ:

(1) y=Φx+η,

where η represents noise. In the second step, the goal is to recover x by solving an optimization problem that balances the accuracy of the data and the sparsity of the signal:

(2) $$\min_{x}\frac{1}{2}{∥y-\Phi x∥}_{2}^{2}+\lambda {∥\Psi x∥}_{1}.$$

Here, Ψ represents the transform basis, and λ controls the trade-off between the reconstruction accuracy and sparsity.

Researchers have developed numerous algorithms to solve this problem. Methods like Orthogonal Matching Pursuit (OMP) [8] and the Iterative Shrinkage-Thresholding Algorithm (ISTA) [9] address the optimization efficiently in many cases. However, computational limitations and reduced accuracy at low sampling rates remain persistent challenges. Algorithms such as the Fast Iterative Shrinkage-Thresholding Algorithm (FISTA) [10] incorporate momentum-based updates to improve convergence speed, significantly enhancing scalability in large-scale settings.

The integration of deep learning with CS has introduced new possibilities. Hybrid frameworks combine traditional optimization methods with neural network-based models to achieve better performance. ISTA-Net [11], for example, adapts ISTA into a trainable neural network, while ADMM-CSNet [12] integrates alternating direction method of multipliers (ADMM) with deep learning for faster and more accurate results. Transformer-based approaches like TransCS [13] utilize attention mechanisms to capture global dependencies effectively. Additionally, architectures based on generative adversarial networks (GANs) [14,15] have been employed to enhance image resolution and recover fine details.

Despite these advancements, computational complexity and trade-offs between speed and accuracy present ongoing difficulties. Transformer-based models, though capable of modeling global features, exhibit quadratic complexity in self-attention mechanisms, limiting their use in real-time applications. Lightweight designs, such as CSNet [16], improve processing speed but often compromise reconstruction quality.

Alternative approaches have emerged to address these shortcomings. The Mamba architecture introduces a selective state-space model that captures local and global dependencies efficiently. Unlike Transformers, which rely on self-attention mechanisms with quadratic complexity $O(n^{2})$, Mamba achieves linear complexity O(n) through a more computationally efficient design. This approach reduces processing overhead and enhances scalability, making it well suited for high-dimensional image data.

Inspired by Mamba’s efficient dependency modeling, this work combines its state-space modeling with the momentum-based optimization of FISTA. The resulting framework achieves faster convergence, improved accuracy, and enhanced adaptability across various reconstruction scenarios.

This study makes the following contributions:

- A novel compressive sensing framework is proposed, integrating Mamba’s state-space modeling and FISTA’s optimization. This design removes the dependence on manually defined hyperparameters such as sensing matrices.
- Improvements in computational efficiency and reconstruction accuracy are demonstrated, with reduced training and inference times.
- Extensive evaluations across multiple datasets highlight the framework’s adaptability and robustness under varying sampling rates and noise levels.

The paper proceeds as follows: Section 2 examines related works in CS and hybrid frameworks. Section 3 describes the proposed framework in detail. Section 4 presents experimental results and comparisons. Section 5 concludes with an outlook on future research.

## 2. Related Work

### 2.1. FISTA

FISTA is an advanced algorithm for solving large-scale optimization problems, particularly in compressive sensing applications. It builds on the traditional Iterative Shrinkage-Thresholding Algorithm (ISTA) by adding momentum-based acceleration, while keeping the simplicity of first-order methods.

The algorithm solves optimization problems of the following form:

(3) $$\min_{x}F\left(x\right)=f\left(x\right)+g\left(x\right)$$

where f(x) is a smooth convex function with a continuous gradient, and g(x) is a convex but possibly non-smooth regularization term. In compressive sensing, this becomes

(4) $$\min_{x}\frac{1}{2}{∥\Phi x-y∥}_{2}^{2}+\lambda {∥x∥}_{1}$$

where Φ is the measurement matrix, y represents the compressed measurements, and λ controls how sparse the solution is.

The key innovation of FISTA is its momentum-based acceleration. In each iteration, the algorithm keeps track of an auxiliary sequence that adds momentum from previous iterations, leading to the following update rules:

(5) $$t_{k+1}=\frac{1+\sqrt{1+4t_{k}^{2}}}{2}$$

(6) $$z_{k}=x_{k}+\frac{t_{k}-1}{t_{k+1}}\left(x_{k}-x_{k-1}\right)$$

Next, the algorithm performs a step called a “proximal gradient step” to adjust the solution:

(7) $$x_{k+1}=\underset{x}{\arg\;\min}\left\{\lambda g\left(x\right)+\frac{L}{2}∥x-\left(z_{k}-\frac{1}{L}\nabla f\left(z_{k}\right)\right){∥}_{2}^{2}\right\}$$

where *L* is the Lipschitz constant of ∇f. For the common $\ell_{1}$ regularization used in compressive sensing, this step simplifies to a soft-thresholding operation:

(8) $${\left[{\mathrm{soft}}_{\lambda /L}\left(v\right)\right]}_{i}=\mathrm{sign}\left(v_{i}\right)\max\{|v_{i}\left|-\lambda /L,0\right\}$$

This combination of momentum acceleration and proximal steps allows FISTA to converge faster, achieving a rate of $O(1/k^{2})$, which is much better than ISTA’s O(1/k) rate. The faster convergence and the algorithm’s efficiency make FISTA especially useful for large-scale compressive sensing tasks, where quick reconstruction is needed.

### 2.2. DL-Based CS

Deep learning has brought substantial advancements to CS by enabling the modeling of complex relationships between measurements and original signals. Methods leveraging deep learning for CS can generally be divided into two categories. The first category combines traditional optimization techniques with neural networks, creating hybrid models that balance interpretability and flexibility. The second category focuses on fully end-to-end architectures, where neural networks directly learn to reconstruct images without relying on prior optimization frameworks.

In the first category, hybrid methods combine the stability of traditional CS algorithms with the efficiency of deep learning. ISTA-Net [11] unfolds the ISTA into a neural network, replacing handcrafted sparsity constraints with learned nonlinear transforms. ADMM-CSNet [12] extends the alternating direction method of multipliers (ADMM) by introducing learnable parameters to accelerate convergence and enhance reconstruction accuracy. AMP-Net unfolds the AMP algorithm into a network framework, addressing noise and reducing blocking artifacts. NeumNet [17] uses the Neumann series to solve inverse problems efficiently but remains susceptible to block effects. Recent approaches, such as TransCS, incorporate Transformer-based architectures to capture global dependencies across image sub-blocks, while DRCAMP-Net [18] combines AMP with residual convolutional layers to expand the receptive field and improve reconstruction performance.

The second category focuses on purely deep learning-based methods, which leverage convolutional neural networks (CNNs) for end-to-end image reconstruction. ReconNet [19] pioneered the use of CNNs for compressive sensing recovery, demonstrating the feasibility of deep learning in this domain. DR2-Net [20] improves reconstruction with residual learning blocks, while DPA-Net [21] employs a dual-path attention mechanism to separately capture structural and texture details. However, the limited receptive field of CNNs restricts their ability to model global dependencies. To address this, methods like MSCRLNet extend the receptive field using multi-scale and residual learning strategies, effectively combining local feature extraction with global modeling.

In addition to these methods, hybrid deep unfolding networks, such as DPC-DUN [22] and LTwIST [23], combine the interpretability of traditional algorithms with the flexibility of deep learning, dynamically optimizing reconstruction paths and eliminating the need for manual parameter tuning.

While these advancements have significantly enhanced image reconstruction, challenges remain in balancing computational efficiency, reconstruction quality, and the ability to capture both local and global features. Recent methods integrating Transformers with CNNs offer promising solutions, paving the way for frameworks like our proposed SSM-Net (the code can be found in Supplementary Material), which further improves the efficiency and accuracy of compressive sensing reconstruction.

### 2.3. Mamba

Mamba is a cutting-edge state-space model (SSM) [24] that has demonstrated remarkable efficiency in modeling long-range dependencies in sequential data. Its key innovation lies in replacing the self-attention mechanism, commonly used in Transformers, with a structured state-space representation. This approach reduces the computational complexity of sequence modeling from quadratic to linear, making Mamba particularly well suited for tasks involving large-scale data. The state-space model underlying Mamba can be expressed as

(9) $$h^{\prime }\left(t\right)=Ah\left(t\right)+Bu\left(t\right),\;y\left(t\right)=Ch\left(t\right)+Du\left(t\right),$$

where $h\left(t\right)\in R^{N}$ is the hidden state, $u\left(t\right)\in R^{N}$ is the input, and $y\left(t\right)\in R^{N}$ is the output. The matrices A, B, C, and D are learnable parameters. To adapt this continuous formulation for deep learning models, it is discretized using zero-order hold (ZOH) as follows:

(10) $$h_{t+1}=e^{A\Delta }h_{t}+\int_{t}^{t+\Delta }e^{A(t+\Delta -\tau )}Bu\left(\tau \right)\;d\tau ,$$

where Δ is the time step.

On this basis, Mamba-2 [25] introduces several architectural improvements that improve both efficiency and performance. Key innovations include a simplified design with parallel parameter generation, an improved state-space dual (SSD) framework for connecting SSM and attention mechanisms, and improved hardware utilization through efficient matrix multiplication operations. The architecture supports larger state sizes (up to 8 times larger than the original Mamba) while maintaining 2–8 times faster computation speed. Mamba-2 also includes an additional normalization layer for improved stability and supports tensor parallelism for better scaling.

The selective information preservation mechanism present in both versions dynamically considers sequence length and input batch size in state computation. Mamba’s scanning method is a key step in converting 2D visual data into 1D sequences. Different scanning methods have different advantages in capturing spatial relationships and contextual information. Global scanning processes the entire image at once and can capture global patterns but may ignore details. Multi-directional selective scanning scans the image from multiple directions and can fully capture spatial information but has higher computational complexity. Spiral scanning expands from the center of the image outward and is suitable for tasks that require full coverage of the image, such as medical imaging and remote sensing. Radial scanning expands from the edge of the image to the center and is suitable for tasks that need to capture details in the central area. The scanning operation facilitates efficient computation of state selection, enabling Mamba to compress historical information into a compact and efficient state representation. This architectural efficiency enables Mamba to be successfully adapted to various computer vision tasks, such as the efficient visual backbone of Vision Mamba [26] and VMamba; the computationally efficient graph processing of Graph/-Mamba [27]; LocalMamba [28], which focuses on the extraction of local features, processing local areas of the image through window selective scanning techniques; and EfficientVMamba [29], which is a lightweight Mamba architecture that reduces computational costs by introducing Atrous selective scanning techniques. Mamba-ND [30] is a multi-dimensional Mamba architecture capable of processing data of arbitrary dimensions. It maintains the linear complexity of SSM by alternating the sequence order, which is suitable for processing high-dimensional data. MambaMixer [31] is a hybrid architecture that combines the advantages of Mamba and Transformer. It enhances the efficiency of sequence modeling through dual token and channel selection mechanisms. Mamba-R [32] introduces register tokens to enhance the focusing ability of feature maps and reduce artifacts in feature maps. PlainMamba [33] is a non-hierarchical Mamba architecture that maintains spatial continuity through continuous 2D scanning. This approach performs well in scenarios that require continuous data processing. Additionally, there are various Mamba-based image segmentation architectures (U-Mamba [34], Swin-UMamba [35], and Mamba-UNet [36]). As an emerging deep learning architecture, Mamba has shown great potential in the field of computer vision through selective state-space models and efficient scanning mechanisms. Its flexible architectural design enables it to adapt to different task requirements, from image classification and video processing to remote sensing and medical image analysis.

## 3. Methods

**Figure 1:**
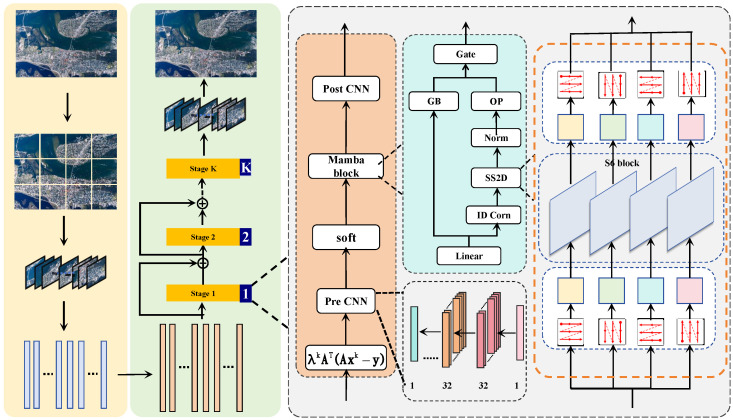
System model.

In this section, we present the proposed framework for efficient image reconstruction. The overall system pipeline is illustrated in Figure 1. The framework consists of three primary components: the sampling module, the reconstruction module, and the loss function design. The sampling module compresses high-dimensional image data into compact measurements, which are then used by the reconstruction module to recover the original image. The reconstruction module includes both initial reconstruction and deep reconstruction stages, iteratively refining the output to achieve high-fidelity results. Finally, the loss function ensures the reconstruction accuracy by balancing fidelity, perceptual similarity, and structural consistency.

### 3.1. Sampling Module

The sampling module is designed to efficiently transform high-dimensional image data into compressed measurements while preserving essential structural information. Given an input image $X\in R^{B\times 1\times H\times W}$, where *B* represents the batch size and *H*, *W* denote the height and width, respectively, we propose a structured block-wise sampling approach that leverages learnable measurement matrices.

The foundation of our sampling mechanism is a learnable measurement matrix $\Phi \in R^{m\times n}$, where m=⌊ρn⌋ with ρ being the target compression ratio and $n=\phi_{\mathrm{size}}^{2}$ denoting the dimensionality of each block. The complete sampling process is detailed in Algorithm 1.
**Algorithm 1** Block-wise Adaptive Sampling Process**Require:** 
Input image $X\in R^{B\times 1\times H\times W}$**Ensure:** 
Compressed measurements y  1:{Initialize measurement matrix}  2:$n\leftarrow \phi_{\mathrm{size}}^{2}$  3:m←⌊ρn⌋  4:$\Phi_{\mathrm{init}}∼N\left(0,{\left(1/n\right)}^{0.5}\right)$  5:$Q_{\mathrm{init}}\leftarrow \Phi_{\mathrm{init}}^{T}$ {Initialize reconstruction matrix}  6:{Block partitioning}  7:**if** $H\;\mathrm{mod}\;\phi_{\mathrm{size}}\neq 0$ or $W\;\mathrm{mod}\;\phi_{\mathrm{size}}\neq 0$ **then**  8:   Apply padding operator B  9:**end if**10:$X_{\mathrm{blocks}}\leftarrow P\left(X\right)$11:{Vectorization}12:$x\leftarrow V(X_{\mathrm{blocks}})$13:{Measurement computation}14:$y\leftarrow \Phi x^{T}$15:**return** y

The sampling process consists of three key transformations. Initially, we define a block partitioning operator $P:R^{B\times 1\times H\times W}\to R^{N\times \phi_{\mathrm{size}}\times \phi_{\mathrm{size}}}$, where *N* represents the total number of blocks. This operator decomposes the input image into non-overlapping blocks:

(11) $$X_{\mathrm{blocks}}=P\left(X\right)$$

Subsequently, we employ a vectorization operator $V:R^{N\times \phi_{\mathrm{size}}\times \phi_{\mathrm{size}}}\to R^{N\times \phi_{\mathrm{size}}^{2}}$ that transforms each block into a vector representation:

(12) $$x=V(X_{\mathrm{blocks}})$$

The final transformation computes the compressed measurements through linear projection:

(13) $$y=\Phi x^{T}$$

A key innovation in our approach is the simultaneous learning of a reconstruction initialization matrix $Q\in R^{n\times m}$, initialized as $Q_{\mathrm{init}}=\Phi_{\mathrm{init}}^{T}$. This dual learning strategy enables joint optimization of the sampling and initial reconstruction processes, leading to the following objective:

(14) $$\min_{\Phi ,Q}{∥X-f_{\mathrm{recon}}\left(Q\Phi x\right)∥}_{2}^{2}$$

where $f_{\mathrm{recon}}\left(\cdot \right)$ represents our reconstruction network.

To handle inputs of any size, we use a padding operator B to adjust the input to the nearest multiple of $\phi_{\mathrm{size}}$. After the experiments, we found that $\phi_{\mathrm{size}}=32$ provides the best balance between speed and reconstruction quality. The adaptive sampling matrices and block-based processing help the framework capture important image features, while keeping the computation simple. This sampling module provides a strong foundation for reducing dimensionality efficiently while preserving critical image structures. It prepares the data for the reconstruction process, which is explained in the next section.

### 3.2. Reconstruction Module

#### 3.2.1. Initial Reconstruction

The image signal is compressed into y through the sampling module, and this y is used as the input to the initial reconstruction, providing the preliminary estimate $x_{\mathrm{init}}$. This estimate forms the basis for subsequent refinement and optimization.

The process begins with a linear operation:

(15) $$x_{\mathrm{init}}=Qy,$$

where $Q\in R^{n\times m}$ is initialized as the transpose of the sampling matrix Φ. Specifically, $Q_{\mathrm{init}}=\Phi^{T}$, ensuring that the initial estimate aligns naturally with the compressed data.

To maintain stability and support effective training, the entries of Q are drawn from a normal distribution:

(16) $$Q_{\mathrm{init}}∼N\left(0,\frac{1}{m}\right),$$

where *m* denotes the dimensionality of the compressed measurements. This initialization strategy promotes stable gradient flow during backpropagation and enables the model to learn meaningful reconstruction patterns.

When the dimensions of the input image do not align with the block size used in the sampling module, padding is applied to adjust the height (*H*) and width (*W*). The required padding values are determined as follows:

(17) $$\begin{array}{cc} h_{\mathrm{pad}} & =\left\{\begin{array}{cc} 0 & \mathrm{if}\;H\;\mathrm{mod}\;\phi_{\mathrm{size}}=0,\\ \phi_{\mathrm{size}}-H\;\mathrm{mod}\;\phi_{\mathrm{size}} & \mathrm{otherwise}, \end{array}\right. \end{array}$$

(18) $$\begin{array}{cc} w_{\mathrm{pad}} & =\left\{\begin{array}{cc} 0 & \mathrm{if}\;W\;\mathrm{mod}\;\phi_{\mathrm{size}}=0,\\ \phi_{\mathrm{size}}-W\;\mathrm{mod}\;\phi_{\mathrm{size}} & \mathrm{otherwise}. \end{array}\right. \end{array}$$

This adjustment ensures consistent processing of all image blocks and retains the spatial relationships present in the original image.

#### 3.2.2. Deep Reconstruction

The initial reconstruction result $x_{\mathrm{init}}$ is used as input to the deep reconstruction module, where it is further improved through iterative optimization and advanced feature modeling. This stage combines momentum-based updates inspired by FISTA with multi-directional selective state-space modeling (SSM). Together, these techniques enable efficient and accurate recovery of high-dimensional image data from compressed measurements. The full process is described in Algorithm 2.
**Algorithm 2** Deep Reconstruction Process**Require:** 
Initial reconstruction $x_{\mathrm{init}}$, compressed measurements y, sampling matrix Φ**Ensure:** 
Final reconstructed image $X_{\mathrm{recon}}$  1:Initialize $x^{\left(0\right)}=x_{\mathrm{init}}$, $t_{1}=1$  2:**for** l=1 to *L* **do**  3:   Compute momentum $t_{l+1}\leftarrow \frac{1+\sqrt{1+4t_{l}^{2}}}{2}$  4:   Update intermediate state $z^{\left(l\right)}\leftarrow x^{\left(l\right)}+\frac{t_{l}-1}{t_{l+1}}\left(x^{\left(l\right)}-x^{\left(l-1\right)}\right)$  5:   Apply gradient correction $z^{\left(l\right)}\leftarrow z^{\left(l\right)}-\eta \Phi^{⊤}\left(\Phi z^{\left(l\right)}-y\right)$  6:   Pre-process features $z^{\left(l\right)}\leftarrow z^{\left(l\right)}-f_{\mathrm{pre}}\left(z^{\left(l\right)}\right)$  7:   Apply selective state-space modeling:  8:   **for** each direction r∈{1,2,3,4} **do**  9:     Compute state evolution $h_{r}^{\left(l\right)}\leftarrow \exp\left(\Lambda \Delta_{r}\right)h_{r}^{\left(l-1\right)}+B_{r}z^{\left(l\right)}\Delta_{r}$10:   **end for**11:   Aggregate results $z^{\left(l\right)}\leftarrow \sum_{r=1}^{4}h_{r}^{\left(l\right)}$12:   Post-process features $x^{\left(l+1\right)}\leftarrow z^{\left(l\right)}-f_{\mathrm{post}}\left(z^{\left(l\right)}\right)$13:**end for**14:Reshape final result $X_{\mathrm{recon}}\leftarrow R\left(x^{\left(L\right)}\right)$15:**return** $X_{\mathrm{recon}}$

In each iteration *l*, the reconstruction follows these steps:

First, momentum-based acceleration is used to speed up convergence. The intermediate state $z^{\left(l\right)}$ is updated as follows:

(19) $$\begin{array}{cc} t_{l+1} & =\frac{1+\sqrt{1+4t_{l}^{2}}}{2}, \end{array}$$

(20) $$\begin{array}{cc} z^{\left(l\right)} & =x^{\left(l\right)}+\frac{t_{l}-1}{t_{l+1}}\left(x^{\left(l\right)}-x^{\left(l-1\right)}\right), \end{array}$$

where $t_{l}$ is a momentum factor initialized as $t_{1}=1$. This step incorporates information from previous iterations to ensure faster convergence and improved reconstruction quality.

Next, to maintain consistency with the compressed measurements y, a gradient descent step is applied to $z^{\left(l\right)}$:

(21) $$z^{\left(l\right)}=z^{\left(l\right)}-\eta \Phi^{⊤}\left(\Phi z^{\left(l\right)}-y\right),$$

where η>0 is a learnable step size parameter. This correction minimizes the data fidelity term, aligning the reconstruction with the measurement constraints.

Before applying state-space modeling, a pre-processing network $f_{\mathrm{pre}}\left(\cdot \right)$ is employed to enhance local image features:

(22) $$z^{\left(l\right)}=z^{\left(l\right)}-f_{\mathrm{pre}}\left(z^{\left(l\right)}\right),$$

where $f_{\mathrm{pre}}\left(\cdot \right)$ is implemented as a series of convolutional layers designed to extract fine-grained image details.

The refined intermediate signal $z^{\left(l\right)}$ is then processed using a multi-directional selective state-space model. This step captures both local and global dependencies by scanning the input in four directions: horizontal, vertical, main diagonal, and secondary diagonal. For each direction *r*, the modeling is defined as

(23) $$\begin{array}{cc} h_{r}^{\left(l\right)} & =\exp\left(\Lambda \Delta_{r}\right)h_{r}^{\left(l-1\right)}+B_{r}z^{\left(l\right)}\Delta_{r}, \end{array}$$

(24) $$\begin{array}{cc} \Delta_{r} & =\phi \left(W_{r}\tau_{r}+b_{r}\right), \end{array}$$

Here, Λ represents the state evolution matrix, $B_{r}$ is the input projection matrix, ϕ(·) is a nonlinear activation function that ensures stability, and $\Delta_{r}$ is the adaptive time step for the *r*-th direction. The outputs from all directions are combined as

(25) $$z^{\left(l\right)}=\sum_{r=1}^{4}h_{r}^{\left(l\right)}.$$

After state-space modeling, a post-processing network $f_{\mathrm{post}}\left(\cdot \right)$ refines the reconstructed signal. This refinement is performed using

(26) $$x^{\left(l+1\right)}=z^{\left(l\right)}-f_{\mathrm{post}}\left(z^{\left(l\right)}\right),$$

where $f_{\mathrm{post}}\left(\cdot \right)$ is a set of learnable convolutional layers designed to remove residual errors and improve the global structure of the signal.

Once *L* iterations are complete, the final reconstructed signal $x^{\left(L\right)}$ is reshaped into its original spatial dimensions:

(27) $$X_{\mathrm{recon}}=R\left(x^{\left(L\right)}\right),$$

where R(·) is the reshaping operator.

This iterative process combines momentum-based optimization, feature extraction, and selective state-space modeling to achieve accurate and high-quality reconstruction. The integration of learnable parameters and adaptive strategies allows the model to handle diverse image structures and varying compression conditions effectively, ensuring both efficiency and accuracy.

### 3.3. Loss Function

To ensure accurate and high-quality reconstruction, we design a loss function that balances pixel-wise fidelity, perceptual quality, and structural consistency.

The MSE loss ensures pixel-wise accuracy by minimizing the $L_{2}$ norm between the reconstructed image $X_{\mathrm{recon}}$ and the ground truth X:

(28) $$L_{\mathrm{MSE}}=\frac{1}{N}{∥X_{\mathrm{recon}}-X∥}_{2}^{2},$$

where *N* is the total number of pixels.

To improve perceptual quality, the SSIM loss measures structural similarity between $X_{\mathrm{recon}}$ and X:

(29) $$L_{\mathrm{SSIM}}=1-\mathrm{SSIM}\left(X_{\mathrm{recon}},X\right),$$

where SSIM(·,·) captures luminance, contrast, and structure.

The edge gradient loss enhances edge preservation by penalizing differences in image gradients:

(30) $$L_{\mathrm{edge}}=\frac{1}{N}\left(∥\nabla_{h}X_{\mathrm{recon}}-\nabla_{h}{X∥}_{1}+{∥\nabla_{v}X_{\mathrm{recon}}-\nabla_{v}X∥}_{1}\right),$$

where $\nabla_{h}$ and $\nabla_{v}$ represent horizontal and vertical gradient operators, respectively.

Finally, the total loss integrates these components, balancing their contributions with weighting factors $\lambda_{\mathrm{SSIM}}$ and $\lambda_{\mathrm{edge}}$:

(31) $$L_{\mathrm{total}}=L_{\mathrm{MSE}}+\lambda_{\mathrm{SSIM}}L_{\mathrm{SSIM}}+\lambda_{\mathrm{edge}}L_{\mathrm{edge}}.$$

This comprehensive loss function ensures faithful reconstruction while preserving structural details and perceptual quality, enabling the model to achieve robust and high-quality results.

## 4. Experimental Results

### 4.1. Experimental Settings

The training and evaluation of SSM-Net use a variety of datasets, including satellite imagery, urban environments, natural scenes, and high-resolution images. The primary training process relies on the BSD500 dataset [37], which consists of 200 training images, 100 validation images, and 200 testing images. From each training image, 200 patches of size 96×96 pixels are extracted, resulting in a total of 100,000 training samples. Data augmentation methods, such as bidirectional flips, rotations, and scaling, are applied to increase image diversity.

To evaluate performance, several benchmark datasets are selected, each addressing specific challenges in image reconstruction:

(1) UCMerced Land Use Dataset: This dataset includes 21 land-use classes, each containing 100 images at a spatial resolution of 0.3 m. The dataset evaluates how well the model processes and distinguishes diverse land cover patterns.

(2) Urban100 [38]: This high-resolution dataset focuses on urban architecture and building facades. It tests the model’s ability to capture detailed structural features in dense urban environments.

(3) BSD100 [39]: A dataset of natural scene images, which features a range of terrain types and vegetation patterns. It provides insight into the model’s generalization capabilities across varied natural landscapes.

(4) Set5 [40]: This benchmark dataset contains high-resolution images designed to evaluate the reconstruction of fine-scale features. It is particularly useful for scenarios where precise detail recovery is critical.

Each dataset presents unique challenges, offering insights into the model’s behavior under different conditions. These datasets also serve as standardized benchmarks, enabling direct comparisons with existing methods such as ISTA-Net+, Csformer [41], AMP-Net, CPP-Net [42], and TransCS. By incorporating diverse structural patterns, textures, and details, the evaluation framework ensures a rigorous assessment of reconstruction performance. The observed results reflect the model’s ability to adapt to various image characteristics, confirming its effectiveness in reconstructing high-quality images under challenging conditions.

All experiments were conducted using PyTorch 1.9.0 on a machine equipped with an Intel^®^ Xeon^®^ 8336 CPU and a GeForce RTX 4090 GPU. To ensure fair comparison, all competing models were trained using the same BSD500 dataset and evaluated under identical conditions across all test datasets.

### 4.2. Comparisons with State-of-the-Art Methods

Table 1 presents comprehensive quantitative comparisons between SSM-Net and current state-of-the-art methods across four benchmark datasets (UCMerced, Set5, Urban100, and BSD100) at various sampling rates (τ). The sampling rate τ is defined as the ratio between the number of measurements *M* and the total number of pixels in the image *N*. Specifically, we define the sampling rate as

$$\tau =\frac{M}{N}$$

This sampling rate controls how much of the image is used during the reconstruction process, with lower values of τ corresponding to higher levels of compression. The experimental results demonstrate that SSM-Net achieves competitive or superior performance compared to existing approaches.

On the UCMerced dataset, which consists of satellite remote sensing imagery, SSM-Net demonstrates competitive performance particularly at lower sampling rates. At τ=0.04, our method achieves a PSNR of 25.28 dB and an SSIM of 0.6959, outperforming both CSformer (25.21 dB/0.6957) and TransCS (25.18 dB/0.6950). The advantage is more pronounced at τ=0.1, where SSM-Net achieves 29.53 dB PSNR and 0.8449 SSIM, surpassing TransCS (29.41 dB/0.8412) and CSformer (29.47 dB/0.8437). At τ=0.25, our method achieves the highest PSNR of 34.71 dB among all compared methods, demonstrating its particular effectiveness in medium-rate compression scenarios for remote sensing applications. While the performance shows some limitations at very high sampling rates (τ=0.5), the strong results in the critical low-to-medium sampling rate range highlight SSM-Net’s practical value for bandwidth-constrained remote sensing scenarios where efficient compression is most needed.

For the Set5 dataset, SSM-Net demonstrates superior performance across the full range of sampling rates. At lower rates (τ=0.01 and 0.04), our method achieves 23.37 dB and 29.32 dB PSNR, respectively, outperforming TransCS (22.98 dB and 29.02 dB). This advantage is maintained through medium rates (τ=0.25 and 0.3) with PSNRs of 37.61 dB and 38.74 dB, and extends to higher rates (τ=0.4 and 0.5) reaching 41.81 dB and 42.72 dB, consistently surpassing both TransCS and CSformer across all compression levels.

In the Urban100 dataset, which contains complex urban structures, SSM-Net shows balanced performance across different sampling rates. While slightly lower than TransCS at τ=0.01 (19.18 dB vs. 19.53 dB), our method demonstrates competitive results at medium rates (τ=0.25 and 0.3) with PSNRs of 30.78 dB and 31.21 dB, and achieves superior reconstruction at higher rates (τ=0.4 and 0.5) with values of 33.34 dB and 34.93 dB, respectively, demonstrating particular effectiveness in preserving architectural details.

On the BSD100 dataset, featuring diverse natural landscapes, SSM-Net exhibits strong performance across sampling rates, with notable advantages at medium to high rates. Starting from τ=0.1 with 27.60 dB PSNR, our method shows progressive improvement through τ=0.25 and 0.3 (31.45 dB and 32.79 dB), culminating in strong high-rate performance at τ=0.4 and 0.5 (34.96 dB and 36.89 dB). This consistent scaling demonstrates our method’s effectiveness in handling varied natural textures across different compression levels.

Table 2 further shows the WS-PSNR and MSSIM comparisons of different image reconstruction methods on the UCMerced, Set5, Urban100, and BSD100 datasets at various sampling rates (τ∈{0.01,0.04,0.1,0.25,0.5}). The results show that SSM-Net can achieve the current state-of-the-art performance on various datasets and sampling rates.

**Figure 2:**
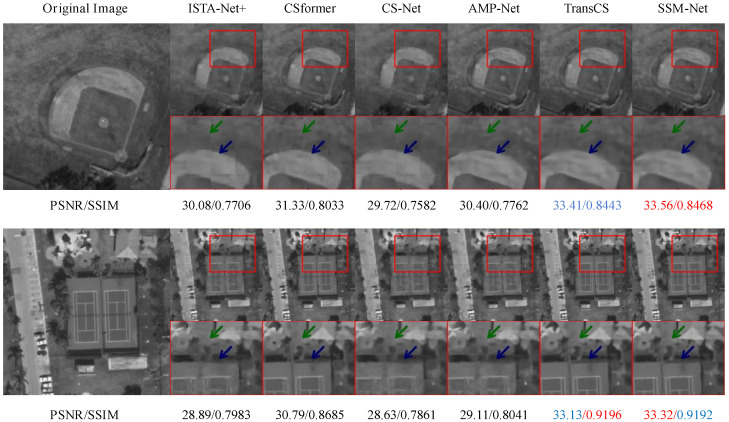
Reconstruction results for satellite sensing images using SSM-Net and other methods. Sampling rates τ are 0.04 for the first row and 0.1 for the second row. Please zoom in for better comparison.

Figure 2 demonstrates the reconstruction results for satellite sensing images. As shown in the detailed regions (highlighted by red boxes), SSM-Net achieves superior reconstruction quality with PSNR/SSIM values of 33.41/0.8443 and 33.32/0.9192 for different sampling rates, effectively preserving both global structure and fine details in remote sensing imagery.

**Figure 3:**
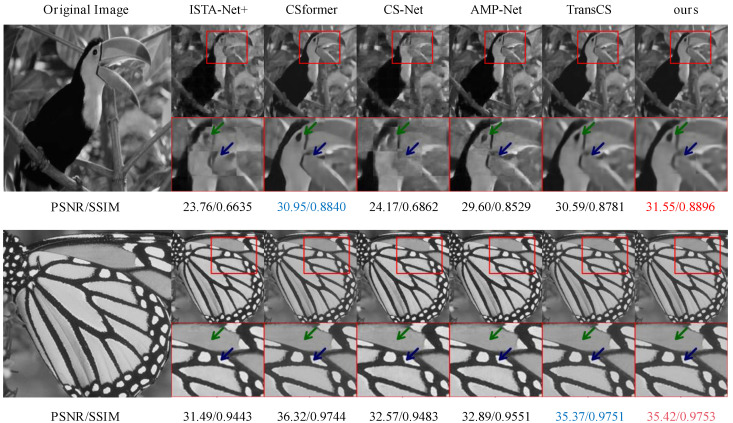
Reconstruction results for high-resolution images using SSM-Net and other methods. Sampling rates τ are 0.04 for the first row and 0.25 for the second row. Please zoom in for better comparison.

Figure 3 provides additional visual comparisons for high-resolution images. The results show that SSM-Net better preserves fine textures and sharp edges, achieving PSNR/SSIM values of 31.55/0.8896 and 35.42/0.9753 for different test cases. This visual quality improvement aligns with the quantitative metrics, confirming our method’s effectiveness in maintaining both structural integrity and local details.

These comprehensive results demonstrate that SSM-Net has achieved state-of-the-art performance, consistently matching or exceeding existing methods across different datasets and sampling rates. The balanced performance across various scenarios, particularly in remote sensing applications, validates our approach of combining momentum-based optimization with efficient feature modeling through the Mamba architecture.

### 4.3. Noise Robustness Analysis

We evaluate our method’s robustness against measurement noise using the BSD100 dataset. Our experiments introduce Gaussian noise with different variances at various sampling rates (τ∈{0.04,0.10,0.25}). Table 3 presents the quantitative comparisons between SSM-Net, AMP-Net, and Csformer under these conditions.

SSM-Net consistently outperforms the competing methods across all noise levels and sampling rates. At low noise (σ=0.001), our method achieves higher PSNR values than both AMP-Net and Csformer, with improvements of up to 0.95 dB at τ=0.04. This advantage remains evident at higher noise levels (σ=0.004), where SSM-Net maintains better reconstruction quality with PSNR values of 22.74 dB, 24.31 dB, and 26.06 dB at sampling rates of 0.04, 0.10, and 0.25, respectively.

**Figure 4:**
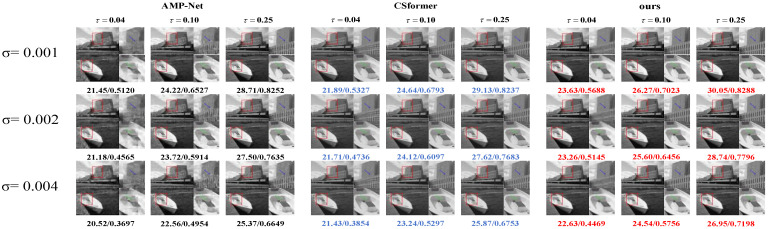
Noise robustness comparison. Visual analysis of different CS methods on images from the BSD100 dataset at sampling rates τ∈0.04,0.10,0.25. Gaussian noise with variances σ∈0.001,0.002,0.004 was introduced. Note the effectiveness in recovering the images.

The visual comparisons in Figure 4 further demonstrate our method’s superior noise handling capabilities. SSM-Net preserves more image details and produces cleaner reconstructions compared to AMP-Net and Csformer, particularly in challenging cases with both high noise levels and low sampling rates. These results confirm that our approach offers enhanced stability and reconstruction accuracy in noisy conditions.

### 4.4. Training Convergence Rate Analysis

**Figure 5:**
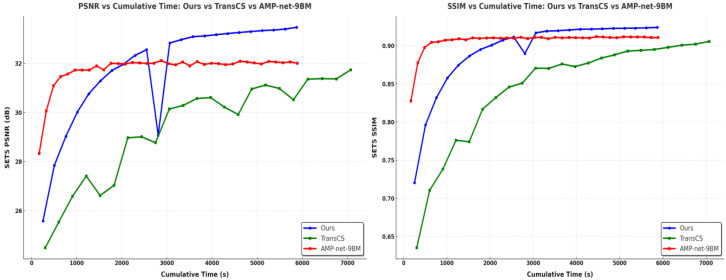
PSNR and SSIM changes in models trained in different ways as training time increases.

We analyze the training convergence speed by comparing the PSNR and SSIM curves of different methods during the training process, as shown in Figure 5. The curves are obtained by evaluating performance on the validation set during training iterations.

Our SSM-Net achieves faster convergence compared to TransCS, reaching stable PSNR and SSIM values within approximately 3000 s. In contrast, TransCS requires nearly 5000 iterations to achieve comparable performance levels. While AMP-Net converges slightly faster, reaching stability at around 2000 s, its final reconstruction quality is notably lower than that of SSM-Net. For example, at sampling rate τ=0.1, SSM-Net achieves a 1.23 dB PSNR improvement over AMP-Net after convergence.

The rapid convergence of SSM-Net can be attributed to two factors: the momentum-based optimization strategy from FISTA and the efficient feature modeling of the Mamba architecture. Specifically, FISTA optimizes the training process by incorporating momentum, which accelerates convergence by utilizing information from previous gradients. This allows the model to make larger, more accurate updates during training, speeding up the process and improving performance.

The Mamba architecture, in contrast to the Transformer-based models, improves convergence by modeling both local and global feature dependencies efficiently. Transformers, while highly effective at capturing global relationships, require extensive computational resources due to their self-attention mechanism, which scales quadratically with the image size. Mamba’s state-space modeling (SSM) addresses this challenge by representing dependencies in a more compact and efficient manner, reducing computational complexity. By utilizing SSM, Mamba effectively captures long-range dependencies without the heavy computational burden typical of Transformers, leading to faster convergence and better performance in terms of both quality and efficiency.

The sudden drop observed in the blue curve (representing SSM-Net) around 2000 s is a notable point. This drop can be explained by the dynamic adjustments the model makes during training. It likely occurs due to the optimization algorithm adapting to the more challenging aspects of the data as the training progresses, where certain features or structures in the image require fine-tuning. This brief decrease is followed by rapid recovery, indicating that the model’s optimization strategy is robust enough to handle such challenges, and it stabilizes quickly. This behavior highlights the balance between exploration and fine-tuning during training, which is an essential aspect of the learning process.

Thus, the combination of FISTA’s momentum-based optimization and Mamba’s state-space modeling contributes significantly to the fast convergence of SSM-Net. The architecture’s efficiency in modeling dependencies and its ability to quickly adapt to complex image features ensure that the network converges faster while maintaining high reconstruction quality.

### 4.5. Complexity Analysis

We analyze the computational complexity of SSM-Net compared to existing methods. All experiments use a standard input size of 256×256 pixels and sampling rate τ=0.1.

**Figure 6:**
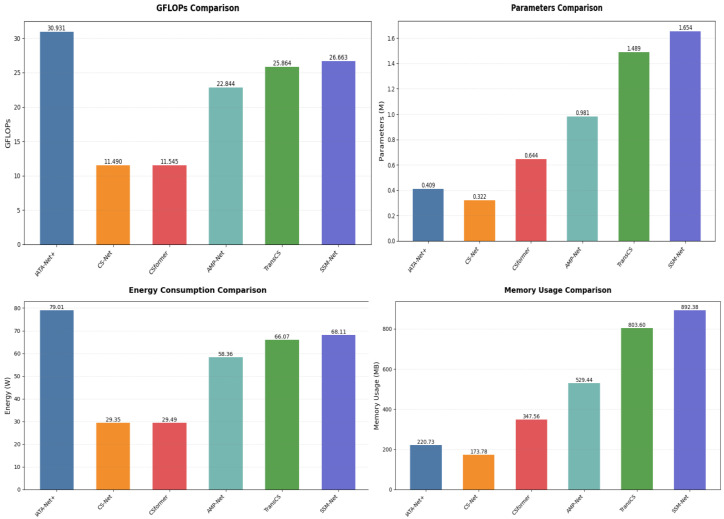
Comparison of GFLOPs, parameters, memory consumption, and energy consumption for a 256×256 pixel image with τ=0.1.

As shown in Figure 6, SSM-Net requires 26.6626 GFLOPs (billion floating-point operations), which falls between lightweight models like CSNet (11.49 GFLOPs) and heavier models like ISTA-Net+ (30.931 GFLOPs). The parameter count of SSM-Net is 1.654 M, similar to that of TransCS (1.489 M). This moderate computational cost enables SSM-Net to achieve superior reconstruction quality while maintaining reasonable efficiency. In terms of energy consumption, SSM-Net requires 68.11 W, which is higher than lightweight models such as CSNet (29.35 W) and CSformer (29.49 W), but still more efficient than models like ISTA-Net+ (79.01 W). Similarly, SSM-Net’s memory usage stands at 892.38 MB, which is significantly higher than CSNet’s 173.78 MB and CSformer’s 347.56 MB, but lower than TransCS (803.60 MB) and ISTA-Net + (220.73 MB). These values highlight the trade-off between higher model performance and the increased computational cost in terms of energy consumption and memory usage.

Table 4 shows inference time comparisons on an RTX 4090 GPU. SSM-Net consistently processes images in approximately 0.0202 s across different sampling rates. This stable processing time demonstrates better scalability compared to methods like CSformer, which shows increasing inference times from 0.0469 to 0.0486 s at higher sampling rates. While CSNet achieves faster inference (0.0078–0.0099 s), it produces lower-quality reconstructions as shown by the PSNR results.

The efficiency of SSM-Net comes from the linear complexity of Mamba’s state-space model and FISTA’s momentum-based optimization. These components work together to provide fast convergence without excessive computational demands. The results show that SSM-Net balances computational cost and reconstruction quality effectively, offering strong performance while maintaining practical processing speeds.

### 4.6. Ablation Studies

**Figure 7:**
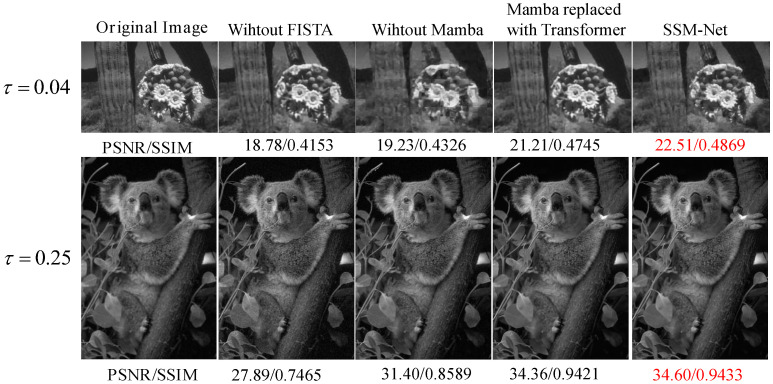
Effectiveness of Mamba and FISTA in SSM-Net reconstruction.

Figure 7 and Table 5 show the results of the ablation study, which evaluates the impact of removing the FISTA algorithm and the Mamba module on image reconstruction quality and computational efficiency. We compare three configurations: SSM-Net (full model), without FISTA, and without Mamba, as well as Mamba replaced with Transformer.

Removing the FISTA algorithm results in a significant reduction in reconstruction quality. At τ=0.25, the PSNR drops from 30.78 dB (SSM-Net) to 27.85 dB (without FISTA), while the runtime improves slightly to 0.0180 s per image. This demonstrates that FISTA is crucial for enhancing both the quality and convergence of the reconstruction.

Similarly, removing the Mamba module also significantly degrades the performance. At τ=0.25, the PSNR decreases from 30.78 dB (SSM-Net) to 28.47 dB (without Mamba). The model also suffers from a loss of important global and local feature modeling, which is essential for high-quality reconstruction. The runtime remains competitive at 0.0184 s per image, but this speed comes at the expense of a noticeable loss in PSNR.

In contrast, the complete SSM-Net model achieves the best balance between reconstruction quality and computational efficiency. At τ=0.25, SSM-Net delivers a PSNR of 30.78 dB, while the runtime is 0.0204 s per image. This shows that the Mamba module plays a key role in improving the reconstruction quality without introducing significant computational overhead.

Finally, replacing Mamba with Transformer brings the PSNR closer to the performance of SSM-Net, reaching 30.12 dB at τ=0.25, compared to 28.47 dB without Mamba. However, the computational time increases significantly to 0.024 s per image. This indicates that although the Transformer configuration achieves PSNR values near SSM-Net, it incurs a substantial increase in runtime due to the quadratic complexity of attention mechanisms, making it less efficient than SSM-Net.

The ablation study demonstrates the critical role of both the Mamba module and FISTA in improving the reconstruction quality. Removing the Mamba module reduces the model’s ability to capture global and local dependencies, as evidenced by the significant drop in PSNR. This shows that Mamba’s capability to model these dependencies is essential for preserving fine image details.

In contrast, FISTA optimization enhances the reconstruction process by accelerating convergence and refining the image quality. When FISTA is removed, the network’s reconstruction is slower, and the model struggles to reach the same level of accuracy. This is reflected in the lower PSNR of “without FISTA” compared to SSM-Net, particularly at higher compression rates, where FISTA’s optimization is most beneficial.

These comparisons clearly demonstrate the benefits of the full SSM-Net model. Removing FISTA or Mamba degrades both the quality and efficiency of the model, while the Transformer-based alternative, although offering PSNR values close to those of SSM-Net, suffers from a substantial increase in runtime. This underscores the importance of Mamba in maintaining high-quality reconstruction with minimal computational cost.

## 5. Conclusions

In this paper, we propose SSM-Net, a new framework for efficient remote sensing image reconstruction based on SSM and deep unfolding techniques. The framework integrates a sampling module for efficient data compression, an initial reconstruction module for fast signal estimation, and a deep reconstruction module that iteratively refines the results. By leveraging FISTA-inspired momentum updates and selective state-space modeling, SSM-Net achieves a balance between reconstruction accuracy, computational efficiency, and fast training convergence.

Comprehensive experiments on standard benchmark datasets demonstrate that SSM-Net offers competitive performance in terms of PSNR and SSIM while maintaining a lightweight architecture and computational efficiency. Although the training process exploits natural image datasets, the framework exhibits strong generalization potential for remote sensing scenarios. Its modular design ensures adaptability and scalability, making it a practical solution for real-world remote sensing applications where storage and transmission constraints are critical.

Looking ahead, we aim to further optimize the proposed framework by incorporating ideas from the Mamba-2 model, particularly the selective state-space decoding SSD methodology. The SSD concept introduces more effective ways of modeling spatial dependencies, which can potentially enhance reconstruction accuracy and robustness. Additionally, we plan to explore domain-specific adaptations of SSM-Net by fine-tuning the framework on large-scale remote sensing datasets, including hyperspectral and SAR images. These advancements will further extend the applicability of SSM-Net, paving the way for its deployment in practical remote sensing systems.

## Figures and Tables

**Table 1:** PSNR (dB) and SSIM comparisons of different methods on datasets Urban100, BSD100, and Set5 at multiple sampling rates (τ∈{0.01,0.04,0.1,0.25,0.3,0.4,0.5}). The highest value is marked in red, and the second highest value is marked in blue.

Datasets	Methods	0.01		0.04		0.10		0.25		0.30		0.40		0.50	
		PSNR	SSIM	PSNR	SSIM	PSNR	SSIM	PSNR	SSIM	PSNR	SSIM	PSNR	SSIM	PSNR	SSIM
UCMerced	ISTA-Net+ (CVPR2018)	17.82	0.4127	21.65	0.5892	25.44	0.7165	29.78	0.8825	31.04	0.9061	33.32	0.9373	35.36	0.9571
	CSNet (TIP2019)	18.89	0.4438	22.69	0.6173	26.17	0.7789	30.76	0.9033	32.52	0.9278	34.83	0.9462	36.47	0.9627
	AMP-Net (TIP2021)	19.12	0.4567	23.74	0.6513	27.90	0.7959	32.55	0.9174	33.44	0.9317	35.26	0.9521	37.65	0.9702
	TransCS (TIP2022)	**21.76**	**0.4836**	25.18	0.6950	29.41	0.8412	34.03	0.9303	**36.12**	**0.9527**	**38.45**	**0.9692**	**40.56**	**0.9785**
	CSformer (TIP2023)	21.52	0.4793	25.21	0.6957	29.47	0.8437	34.57	0.9387	35.97	0.9513	38.21	0.9675	40.23	0.9771
	CPP-Net (CVPR2024)	21.40	0.4783	25.14	0.6908	29.46	0.8427	34.23	0.9328	35.55	0.9498	37.11	0.9629	39.31	0.9758
	SSM-Net (Ours)	21.38	0.4705	**25.28**	**0.6959**	**29.53**	**0.8449**	**34.71**	**0.9398**	35.89	0.9511	36.12	0.9568	37.33	0.9613
Set5	ISTA-Net+ (CVPR2018)	20.25	0.5608	23.42	0.6287	28.47	0.8309	34.02	0.9188	35.38	0.9397	37.44	0.9573	39.25	0.9689
	CSNet (TIP2019)	20.15	0.5447	27.12	0.7988	31.07	0.8925	35.89	0.9473	37.25	0.9473	38.91	0.9611	40.74	0.9691
	AMP-Net (TIP2021)	20.45	0.5563	27.25	0.8065	31.43	0.8977	36.25	0.9514	37.82	0.9583	39.55	0.9694	41.48	0.9756
	TransCS (TIP2022)	22.98	0.6287	29.02	0.8317	32.74	0.9235	37.26	0.9625	38.53	0.9693	41.40	0.9773	42.42	0.9846
	CSformer (TIP2023)	21.84	0.5892	29.27	0.8239	33.04	0.9243	37.04	0.9583	38.44	0.9614	40.62	0.9723	42.37	0.9793
	CPP-Net (CVPR2024)	22.63	0.6214	29.19	0.8259	32.64	0.9240	37.12	0.9592	38.24	0.9598	40.90	0.9746	42.25	0.9769
	SSM-Net (Ours)	**23.37**	**0.6311**	**29.32**	**0.8377**	**33.17**	**0.9250**	**37.61**	**0.9647**	**38.74**	**0.9712**	**41.81**	**0.9796**	**42.72**	**0.9849**
Urban100	ISTA-Net+ (CVPR2018)	15.23	0.4127	19.65	0.5351	23.44	0.7165	28.78	0.8825	30.04	0.9061	32.32	0.9373	34.36	0.9571
	CSNet (TIP2019)	15.89	0.4438	19.69	0.5973	23.17	0.7789	28.76	0.9033	29.52	0.9278	32.83	0.9462	33.47	0.9627
	AMP-Net (TIP2021)	16.12	0.4567	20.74	0.6013	23.90	0.7859	29.55	0.9174	30.44	0.9317	33.26	0.9521	34.65	0.9702
	TransCS (TIP2022)	**19.53**	**0.5104**	22.30	0.6938	**25.87**	**0.8334**	30.46	0.9215	**31.47**	**0.9446**	**33.49**	**0.9621**	34.58	0.9711
	CSformer (TIP2023)	18.92	0.4893	**22.57**	0.6781	25.27	0.8213	30.57	0.9287	31.07	0.9313	33.21	0.9575	34.23	0.9721
	CPP-Net (CVPR2024)	18.83	0.4856	22.19	0.6759	25.32	0.8255	30.22	0.9269	31.17	0.9358	33.16	0.9549	34.60	0.9699
	SSM-Net (Ours)	19.18	0.4992	21.79	0.6857	25.43	0.8293	**30.78**	**0.9398**	31.21	0.9372	33.34	0.9583	**34.93**	**0.9725**
BSD100	ISTA-Net+ (CVPR2018)	17.45	0.4234	22.21	0.5397	24.89	0.6837	28.83	0.8379	29.92	0.8673	31.77	0.9063	33.52	0.9357
	CSNet (TIP2019)	18.23	0.4567	23.77	0.6497	26.31	0.7714	30.04	0.8997	30.69	0.9135	32.94	0.9299	34.96	0.9478
	AMP-Net (TIP2021)	18.67	0.4623	24.04	0.6537	26.16	0.7688	30.13	0.9002	30.88	0.9142	33.24	0.9379	35.43	0.9517
	TransCS (TIP2022)	22.26	0.4848	24.68	0.6633	**27.76**	**0.7945**	**31.54**	**0.9031**	32.27	0.9215	34.52	0.9499	36.52	**0.9671**
	CSformer (TIP2023)	**22.42**	**0.4892**	24.96	**0.6709**	26.54	0.7749	30.75	0.9022	31.54	0.9189	34.21	0.9443	35.91	0.9589
	CPP-Net (CVPR2024)	22.15	0.4836	24.78	0.6684	27.38	0.7895	30.95	0.9012	31.97	0.9207	34.35	0.9478	36.24	0.9584
	SSM-Net (Ours)	21.87	0.4797	**25.12**	0.6690	27.60	0.7903	31.45	0.8910	**32.79**	**0.9237**	**34.96**	**0.9522**	**36.89**	0.9577

**Table 2:** WS-PSNR (dB) and MSSIM comparisons of different methods on datasets Urban100, BSD100, and Set5 at multiple sampling rates (τ∈{0.01,0.04,0.1,0.25,0.5}). The highest value is marked in red, and the second highest value is marked in blue.

Datasets	Methods	0.01		0.04		0.10		0.25		0.50	
		WS-PSNR	MSSIM	WS-PSNR	MSSIM	WS-PSNR	MSSIM	WS-PSNR	MSSIM	WS-PSNR	MSSIM
UCMerced	ISTA-Net+ (CVPR2018)	16.03	0.4457	18.18	0.6079	21.88	0.7351	27.69	0.8913	33.28	0.9565
	CSNet (TIP2019)	17.11	0.4998	20.12	0.6354	23.98	0.7972	28.63	0.9121	34.39	0.9611
	AMP-Net (TIP2021)	17.37	0.5139	20.19	0.6698	24.32	0.8142	30.48	0.9259	35.57	0.9694
	TransCS (TIP2022)	19.87	0.5321	22.21	0.7621	26.92	0.8922	31.97	0.9487	38.41	0.9881
	CSformer (TIP2023)	19.62	0.5308	22.54	0.7682	27.18	0.8957	32.43	0.9572	38.37	0.9868
	CPP-Net (CVPR2024)	18.48	0.5271	22.13	0.7607	27.01	0.8937	32.05	0.9502	38.18	0.9849
	SSM-Net (Ours)	18.87	0.5274	22.67	0.7690	27.26	0.8965	32.49	0.9590	36.31	0.9778
Set5	ISTA-Net+ (CVPR2018)	16.68	0.5791	18.85	0.6469	26.89	0.8487	31.95	0.9271	35.18	0.9579
	CSNet (TIP2019)	17.59	0.5629	19.92	0.8161	29.49	0.9108	33.81	0.9556	36.24	0.9662
	AMP-Net (TIP2021)	17.88	0.5746	23.68	0.8239	29.86	0.9159	34.18	0.9597	36.41	0.9688
	TransCS (TIP2022)	19.32	0.6552	24.97	0.8784	30.18	0.9479	35.19	0.9708	37.54	0.9745
	CSformer (TIP2023)	19.12	0.6542	25.08	0.8776	30.47	0.9515	34.97	0.9666	37.91	0.9793
	CPP-Net (CVPR2024)	19.27	0.6521	24.65	0.8749	30.07	0.9427	34.95	0.9675	37.42	0.9762
	SSM-Net (Ours)	19.96	0.6589	25.17	0.8798	30.64	0.9522	34.83	0.9728	37.70	0.9788
Urban100	ISTA-Net+ (CVPR2018)	13.65	0.4309	17.08	0.5534	20.87	0.7348	26.71	0.8909	32.29	0.9504
	CSNet (TIP2019)	14.33	0.4611	17.12	0.6156	21.59	0.7972	26.68	0.9117	31.40	0.9520
	AMP-Net (TIP2021)	14.57	0.4742	18.17	0.6196	22.33	0.8429	27.48	0.9257	31.58	0.9595
	TransCS (TIP2022)	16.85	0.5279	19.98	0.7618	24.29	0.8991	28.39	0.9298	32.16	0.9704
	CSformer (TIP2023)	15.43	0.5197	19.72	0.7554	22.98	0.8821	28.49	0.9370	32.51	0.9714
	CPP-Net (CVPR2024)	15.24	0.5182	19.01	0.7532	23.02	0.8832	28.05	0.9352	31.84	0.9689
	SSM-Net (Ours)	15.89	0.5202	19.15	0.7544	23.11	0.8839	27.29	0.9377	31.78	0.9629
BSD100	ISTA-Net+ (CVPR2018)	15.88	0.4417	17.64	0.5581	20.32	0.7019	24.78	0.8462	30.45	0.9449
	CSNet (TIP2019)	16.66	0.4751	20.20	0.6680	22.74	0.7897	26.81	0.9080	31.89	0.9571
	AMP-Net (TIP2021)	17.10	0.4806	20.42	0.6720	22.59	0.7871	27.06	0.9085	31.36	0.9510
	TransCS (TIP2022)	18.69	0.5642	21.11	0.7416	25.19	0.8572	28.23	0.9214	34.45	0.9699
	CSformer (TIP2023)	18.85	0.5666	21.39	0.7492	24.34	0.8541	27.89	0.9199	33.84	0.9682
	CPP-Net (CVPR2024)	18.58	0.5607	21.21	0.7423	24.18	0.8478	27.84	0.9185	33.17	0.9677
	SSM-Net (Ours)	18.49	0.5621	21.47	0.7462	24.56	0.8552	27.92	0.9182	32.53	0.9583

**Table 3:** PSNR (dB) and SSIM comparisons on BSD100 with different noise levels σ and various sampling rates τ.

σ	τ	AMP-Net		Csformer		SSM-Net	
		PSNR	SSIM	PSNR	SSIM	PSNR	SSIM
0.001	0.04	23.28	0.5475	24.12	0.5483	**24.23**	**0.5509**
	0.10	25.32	0.6558	26.21	0.6722	**26.38**	**0.6760**
	0.25	27.73	0.7930	28.54	0.7932	**28.75**	**0.7946**
0.002	0.04	22.59	0.4879	23.41	0.4923	**23.54**	**0.4957**
	0.10	24.17	0.6052	25.31	0.6198	**25.44**	**0.6241**
	0.25	27.23	0.7583	27.33	0.7512	**27.46**	**0.7537**
0.004	0.04	20.56	0.4317	22.56	0.4246	**22.74**	**0.4262**
	0.10	22.53	0.5289	24.18	0.5312	**24.31**	**0.5331**
	0.25	25.41	0.6825	25.89	0.6943	**26.06**	**0.6972**

**Table 4:** Time consumption (in seconds) of different methods under various compression rates τ on GPU: RTX 4090.

Methods	GPU Time Consumption (s)					Platform
	τ = 0.10	τ = 0.25	τ = 0.30	τ = 0.40	τ = 0.50	
ISTA-Net+	0.0227	0.0232	0.0238	0.0241	0.0247	RTX 4090
CSNet	0.0078	0.0084	0.0089	0.0095	0.0099	
CSformer	0.0469	0.0471	0.0476	0.0480	0.0486	
AMP-Net	0.0165	0.0177	0.0181	0.0189	0.0194	
TransCS	0.0241	0.0245	0.0247	0.0251	0.0257	
SSM-Net	0.0201	0.0203	0.0202	0.0203	0.0203	

**Table 5:** Comparisons of PSNR results (dB) and GPU runtime on 4090 for different methods with an input image of pixel size 256 × 256.

Methods	τ=0.10		τ=0.25		τ=0.30	
	PSNR (dB)	GPU Time (s)	PSNR (dB)	GPU Time (s)	PSNR (dB	GPU Time (s)
Without Mamba	26.58	0.0182	28.47	0.0184	29.84	0.0185
Without FISTA	25.40	0.0175	27.85	0.0180	29.10	0.0182
Mamba replaced with Transformer	28.74	0.0234	30.12	0.024	31.89	0.025
SSM-Net	29.32	0.0201	30.78	0.0204	32.45	0.0207

## Data Availability

The original contributions presented in this study are included in the article/Supplementary Material.

## Supplementary Materials

The following supporting information can be downloaded at: https://www.mdpi.com/article/10.3390/s25041026/s1.
